# Development, refinement, and characterization of a nonhuman primate critical care environment

**DOI:** 10.1371/journal.pone.0281548

**Published:** 2023-03-17

**Authors:** Joseph D. Bozzay, Patrick F. Walker, Rex E. Atwood, Robert W. DeSpain, William J. Parker, Daniel S. Chertow, John A. Mares, Crystal L. Leonhardt, Eric A. Elster, Matthew J. Bradley

**Affiliations:** 1 Department of Surgery, Uniformed Services University of the Health Sciences and Walter Reed National Military Medical Center, Bethesda, Maryland, United States of America; 2 Department of Regenerative Medicine, Naval Medical Research Center, Silver Spring, Maryland, United States of America; 3 Emerging Pathogens Section, Critical Care Medicine Department, Clinical Center, National Institutes of Health, Bethesda, Maryland, United States of America; 4 Laboratory of Immunoregulation, National Institute of Allergy and Infectious Diseases, National Institutes of Health, Bethesda, Maryland, United States of America; University of Western Ontario, CANADA

## Abstract

**Background:**

Systemic inflammatory response remains a poorly understood cause of morbidity and mortality after traumatic injury. Recent nonhuman primate (NHP) trauma models have been used to characterize the systemic response to trauma, but none have incorporated a critical care phase without the use of general anesthesia. We describe the development of a prolonged critical care environment with sedation and ventilation support, and also report corresponding NHP biologic and inflammatory markers.

**Methods:**

Eight adult male *rhesus macaques* underwent ventilation with sedation for 48–96 hours in a critical care setting. Three of these NHPs underwent “sham” procedures as part of trauma control model development. Blood counts, chemistries, coagulation studies, and cytokines/chemokines were collected throughout the study, and histopathologic analysis was conducted at necropsy.

**Results:**

Eight NHPs were intentionally survived and extubated. Three NHPs were euthanized at 72–96 hours without extubation. Transaminitis occurred over the duration of ventilation, but renal function, acid-base status, and hematologic profile remained stable. Chemokine and cytokine analysis were notable for baseline fold-change for Il-6 and Il-1ra (9.7 and 42.7, respectively) that subsequently downtrended throughout the experiment unless clinical respiratory compromise was observed.

**Conclusions:**

A NHP critical care environment with ventilation support is feasible but requires robust resources. The inflammatory profile of NHPs is not profoundly altered by sedation and mechanical ventilation. NHPs are susceptible to the pulmonary effects of short-term ventilation and demonstrate a similar bioprofile response to ventilator-induced pulmonary pathology. This work has implications for further development of a prolonged care NHP model.

## Introduction

Trauma remains a leading cause of preventable death, with consequences extending beyond the acute insult of initial injury [1, 2]. The systemic inflammatory response following traumatic injury contributes to the pathophysiology of complications such as thromboembolism, infection, and organ dysfunction which leads to increased morbidity and mortality [1, 3–11]. This complex dysregulated response is not completely understood, but further characterization may lead to the identification of targets for therapeutic intervention which may mitigate the systemic inflammatory effects of injury [3–5, 10–13].

Nonhuman primates (NHPs) demonstrate similar responses to trauma that are comparable to humans, and injury models have recently been described [3–5, 10, 13–19]. While models have been developed for sepsis, no traumatic NHP study has characterized the prolonged inflammatory response and associated complications after injury, particularly with the inclusion of a clinically relevant critical care environment with mechanical ventilatory support [20, 21].

The systemic effects of extended ventilation and sedation in a nonhuman primate without traumatic injury are not known. Our group has previously developed and reported clinically relevant traumatic NHP models, but these did not incorporate a prolonged critical care phase [3–5, 22]. We subsequently designed and implemented a NHP intensive care unit (ICU) to provide mechanical ventilatory and critical care support for at least 48 hours without the use of general anesthesia. We briefly describe our methodology and model development, and also report the bioprofiles of the first eight NHPs that were used for this study.

## Methods

### Animals

The study protocol was reviewed and approved by the Walter Reed Army Institute of Research/Naval Medical Research Center (WRAIR/NMRC) Institutional Animal Care and Use Committee. This study was approved and carried out in compliance with all applicable Federal regulations governing the protection of animals in research, including the National Institutes of Health guide for the care and use of Laboratory animals (NIH Publications No. 8023, revised 1978). Eight adult male (8.6 kg ± 0.6) *rhesus macaques* with an average age of 5.8 ± 0.5 years were utilized. NHPs were quarantined for at least 45 days to acclimate to the animal facility. During that time, they were allowed free access to feed and water, but oral nutrition was withheld the night prior the experiment. Animals had visual contact with other animals in the room (3x3x6 foot cages) in addition to continued environmental enrichment strategies as outlined in our veterinary protocols and IACUC policies. Enrichment strategies included avenues and objects for sensory (audio and visual), cognitive, and motor manipulation, as well as physical habitat changes, such as a roaming area. Animals were allowed free access to food and water prior to the experiment.

### Pre-ICU phase

Animals were sedated with Telazol (4–8 mg/Kg) and underwent mask ventilation with Isoflurane 4–5%/0.21 FiO2 to induce general anesthesia. Intravenous access was established. Animals were then intubated with direct laryngoscopy using uncuffed 4 Fr endotracheal tubes. End-tidal carbon dioxide (EtCO2) and chest radiograph confirmed proper placement of the endotracheal tube. A foley catheter, pulse-oximeter probe, electrocardiac leads, biceps blood pressure cuff, orogastric tube, and rectal probe were placed and used for continuous monitoring throughout the entire study.

**Fig 1:**
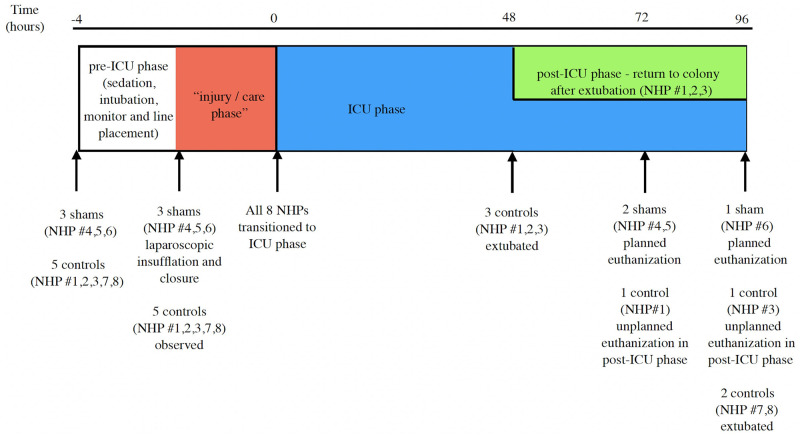
Time schematic of the experimental model.

Eight successive NHPs were used to pilot the development and tailoring of the ICU phase. Of these, three NHPs (NHP 4–6) underwent “sham” surgery in accordance with the next phases of the protocol. The purpose of a “sham” operation was to simulate the operative timing and rehearsal of steps of an injury phase, without actually causing any injury or performing any organ handling. These three sham NHPs underwent laparoscopic insufflation and desufflation of the abdomen without organ manipulation shortly after intubation to simulate the performance of laparoscopic induced injuries (liver and colon) as previously described in our model [4]. This was followed by sharp midline incision and suture closure two hours later prior to the start of the ICU phase to mimic prolonged expeditionary care and subsequent repair of these injuries (Fig 1).

### ICU phase

The animals were then delivered to the ICU suite and transitioned from general anesthesia to intravenous and intramuscular sedation and placed on a Drager Apollo Anesthesia Workstation (Draeer Medical Inc, Telford, Pa). They remained on sedation for the remainder of the experiment until extubation. The ventilator was initiated with a volume-control mode with tidal volume 7–10 ml/kg and respiratory rates of 15–30 breaths per minute. FiO2 was weaned to maintain SpO2 > 90%, and respiratory rate and tidal volume was adjusted to maintain pCO2 between 35–42 mmHg. Peak expiratory pressure (PEEP) was set at 5 cmH2O and peak inspiratory pressures did not exceed 15 cmH2O. Protocol sedative and analgesic medications are shown in Table 1. Sedation and analgesia regimens were chosen and titrated as needed to maintain comfort (normal physiologic vital signs, minimal independent movement). The original regimen was propofol and fentanyl, but this regimen was subsequently changed to alfaxalone and fentanyl. Animals were allowed to overbreathe the ventilator or demonstrate signs of deep sedation (e.g. lack of purposeful movement) if they showed no signs of agitation or vital derangements. Maintenance crystalloid fluid was administered to maintain urine output > 1 ml/kg/hr.

**Table 1:** Sedation and analgesia regimens and doses.

Drug Name (Route)	Dosing Range
Propofol (IV)	2 mg/kg IV bolus with 0.1–0.5 mg/kg/min continuous rate
Dexmedetomidine (IV)	0.4–1 ug/kg/hr
Telazol (IM)	4–8 mg/kg every 4 hours as needed IM
Ketamine (IV)	10–20 mg/kg every 4 hours as needed
Fentanyl (IV)	18.75–50 mcg/hr
Alfaxalone (IV)	25–50 mcg/hr

Shortly after intubation, surgical cutdown was performed to place a sterile arterial line into the right femoral artery using a 22-gauge angio-catheter (Cordis, Johnson & Johnson, New Brunswick NJ). This was connected to a hemodynamic monitoring system (Philips IntelliVue MP70, Philips Electronics North America Corporation, Andover MA) for continuous monitoring of arterial pressures and blood draws. This line was flushed every hour with low volume heparinized saline to keep the arterial catheter patent. A 4Fr double lumen pediatric central venous catheter (Arrow International, Reading PA) was also surgically placed into the right femoral vein. A tunneled 6Fr vascular access port (PORT-A-CATH, Smiths-Medical, Dublin, OH) was placed into the left femoral vein and tunneled to the flank for long-term venous access.

A trained animal care handler and physician remained with the animal for the entirety of the ICU phase to deliver dynamic care and monitoring. The animals were rotated every 2 hours to prevent decubitus ulcers and chlorhexidine oral care was provided every 2–4 hours. Sterile ophthalmic ointment was applied to the eyes every 6 hours to prevent corneal ulcers and eye dryness. Warm blankets and a warming system were used to maintain normothermia. The head and upper body of the NHP was elevated with padding. The environment was dimmed during normal vivarium sleep times. Scheduled in-line suctioning was used to clear the endotracheal tube of any secretions every four hours, and oropharyngeal suctioning was also performed on this interval. The orogastric tube was maintained. No vasopressors were used during this study. Enteral feeds were initially administered to the “sham” NHPs starting 24 hours after the start of the ICU phase. However, high residuals were noted within 24 hours of tube feed initiation, so the enteral feeds were stopped, and dextrose was added to the crystalloid infusion.

### Post-ICU phase

Five of the eight NHPs (NHP 1,2,3,7,8) were survived and intentionally extubated. Extubation criteria included spontaneous respiratory drive > 15 breaths/min while sedated, with tidal volumes of at least 8 ml/kg. The first three control NHPs (1,2,3) were extubated after 48 hours of ventilation and returned to colony housing, but two experienced respiratory complications at subsequent planned sedation attempts for examination and laboratory draws and required unplanned euthanasia at 72 and 96 hours, respectively (NHP 1, 3). The final two controls (NHP 7, 8) were extubated after 96 hours of ventilation and were eventually returned to their colony, along with NHP 2. The three sham NHPs (NHP 4,5,6) were not extubated and were euthanized at the end of their ICU phases without ventilation liberation.

### Laboratory and tissue analysis

Blood samples were collected at 0, 1, 2, and 4 hours and then daily for quantitative laboratory analysis (arterial blood gas, complete blood count, chemistry, ROTEM, systemic inflammatory mediators/danger signaling molecules). Serum profiles for a panel of cytokines and chemokines was determined using multianalyte bead-based profiling (Luminex; NHP 14-plex; Millipore, Billerica, MA). Blood samples were also collected as needed if clinically indicated. Sample rationing or loss of blood samples contributed to nonuniformity amongst the individual NHPs. NHP 7 and 8 did not undergo any cytokine or ROTEM testing, and were monitored primarily with point-of-care testing with the GEM ^®^ Premier 4000 analyzer. A chest radiograph was obtained every morning to confirm endotracheal tube positioning and absence of acute lung pathology.

A complete necropsy was performed immediately after death to include gross examination of all hollow and solid organs, skeletal muscle, and major vasculature. Representative sections of these organs underwent histopathologic evaluation by a skilled veterinary pathologist.

### Statistical analysis

Differences were described Student’s t- test for continuous variables. Significance was defined as a p-value of <0.05. Statistical analysis was completed using SAS version 9.4 (SAS, Cary, NC). All data are presented as mean ± standard error of the mean (SEM). Cytokine data is reported as fold change, based on fluorescence value [23].

## Results

The initial three control NHPs (NHP 1,2,3) survived 48 hours of planned ventilation and were extubated and returned to their housing unit. Two NHPs (NHP 1, 3) were euthanized later for respiratory distress while in the housing unit. Necropsy revealed aspiration and necrotizing bronchopneumonia, respectively. The subsequent three “sham” NHPs (NHP 4,5,6) remained on the ventilator until planned euthanasia and were never extubated and returned to the housing unit. The final two controls (NHP 7,8) survived 96 hours of ventilation.

Two NHPs (NHP 1,3) required euthanasia after they were sedated for examination and lab draw in their colony unit for worsening pulmonary status each at about 72–80 hours. Necropsy revealed aspiration and necrotizing bronchopneumonia in both NHPs, with one suffering multifocal acute pulmonary emboli, which likely originated from a thrombosed central line site.

All NHPs remained hemodynamically normal and stable during the study, except for the two NHPs that acutely decompensated after extubation, requiring unplanned euthanasia. Vasoactive or cardiac medications were used to influence cardiovascular hemodynamics only during induction phase for intubation and experiment initialization. Circadian rhythms, care, adjustment of sedation and analgesic medications, or care tasks largely contributed to any observed fluctuation. Any hypotension was usually attributable to sedation regimen adjustments, and responded to medication titration or small fluid boluses.

**Fig 2:**
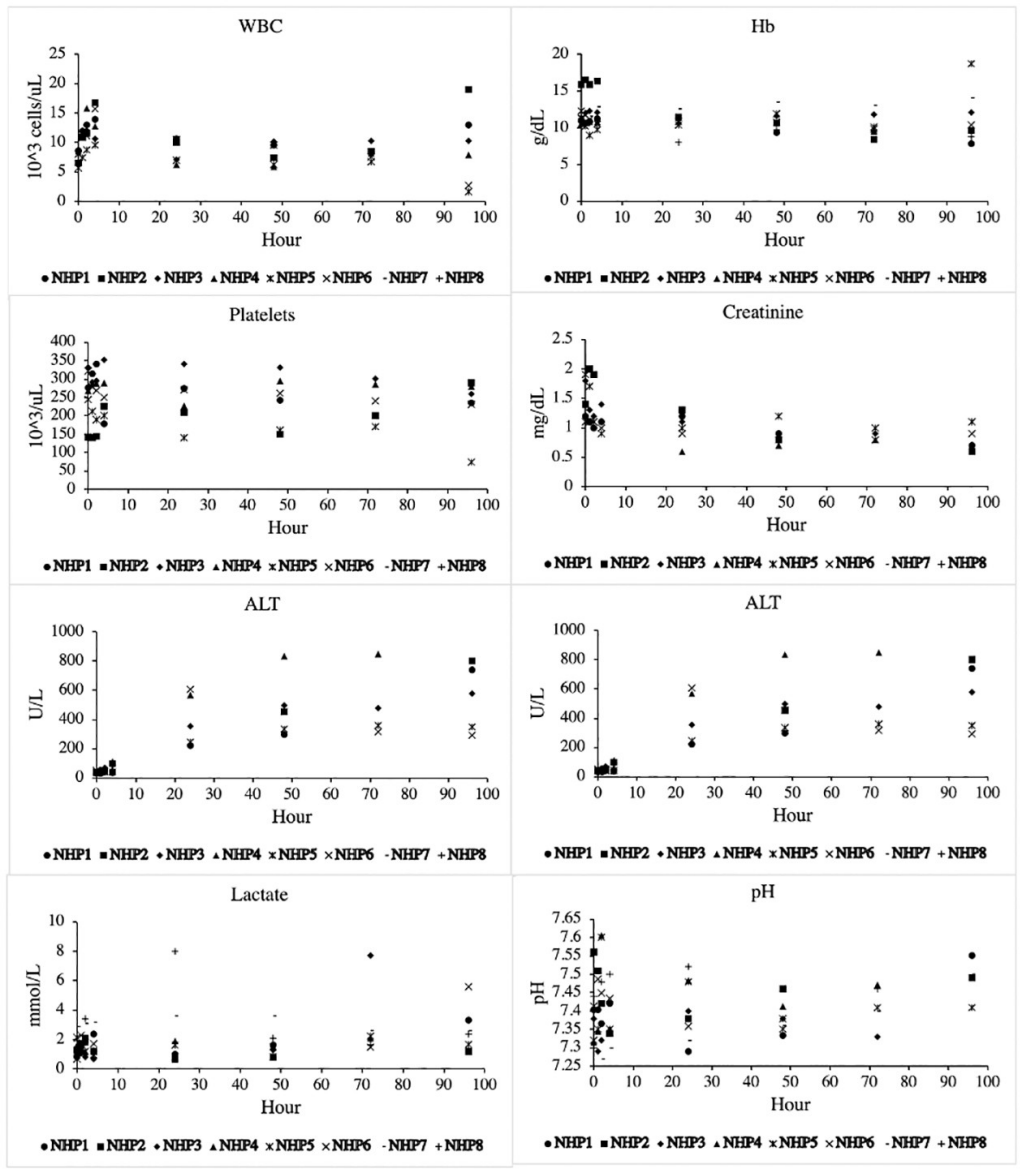
Selected hematologic and chemistry results. * denotes statistical significance from baseline values (p < 0.05).

Results from complete blood counts, chemistries, and arterial blood gases are shown in Fig 2. Rationing of blood draws or loss of blood samples contributed to nonuniformity amongst the individual NHPs. White blood cell count peaked early during the experiment but downtrended to normal throughout the ICU period. A gradual downtrend of hemoglobin (Hb) was usually observed, but only blood loss occurred from the described blood draws (using pediatric tubes) or line placement. The greatest change was demonstrated by alanine aminotransferase (ALT) and aspartate aminotransferase (AST), which increased throughout the experiment. Creatinine (Cr) remained relatively normal with a slight downtrend. Hematologic acid-base status remained stable and physiologic, with lactate and pH staying relatively constant unless clinical respiratory decompensation was observed.

**Fig 3:**
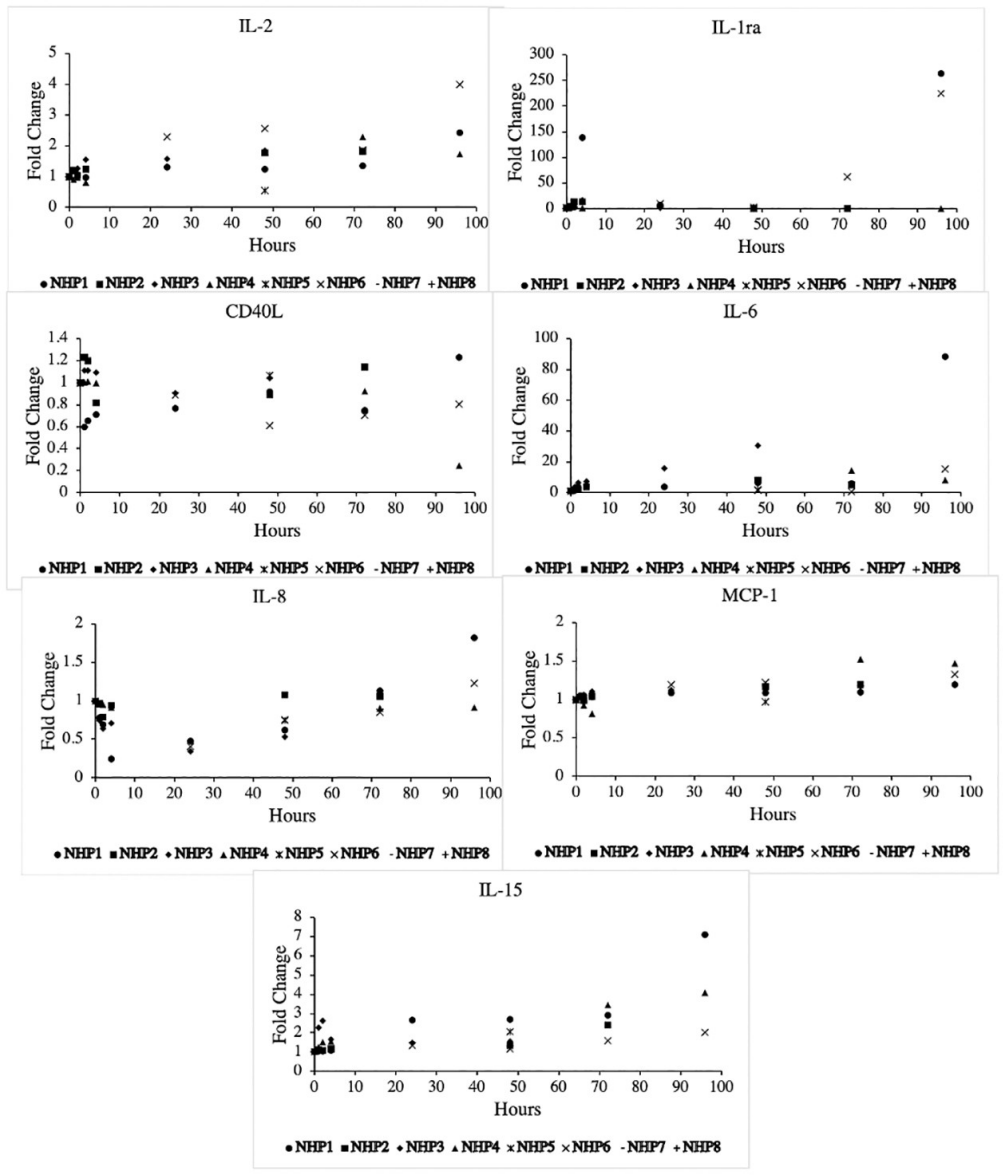
Selected biomarker results.

The systemic inflammatory response manifested by selected serum cytokines and chemokines are shown in Fig 3 and Table 2. Several cytokines and chemokines were only intermittently detected during the experiment and so no fold-change trends were tracked for select markers. These markers fluctuated near background fluorescence, so it was not possible to distinguish a detectable change. Notably, IL-6 and IL-1ra demonstrated rapid early peaks that downtrended and stabilized unless clinical deterioration was observed. Just prior to unplanned euthanasia for acute respiratory decompensation, such as aspiration, GCSF, IL-1b, IL-1ra, IL-10, and IL-6 rapidly increased in the affected NHPs (NHP 1,3).

**Table 2:** Fold changes for various biomarkers.

Marker	Average Peak Fold change (SEM)	Average fold change prior to unplanned euthanasia
IL-2	1.72 (0.29)	3.21
IL-1ra	42.7 (32)	244
CD40	0.99 (0.12)	1.01
IL-6	9.73 (4.88)	52
IL-8	1.01 (0.08)	1.52
MCP-1	1.14 (0.03)	1.26
IL-15	2.3 (0.39)	4.56
IL-10	-	525
TNF-alpha	-	1.3
G-CSF	-	1074
IL-1b	-	1171
IL-12	-	
IL-17a	-	
IL-4	-	
IFN-y	-	3.1

- fluctuated near baseline fluorescence.

**Fig 4:**
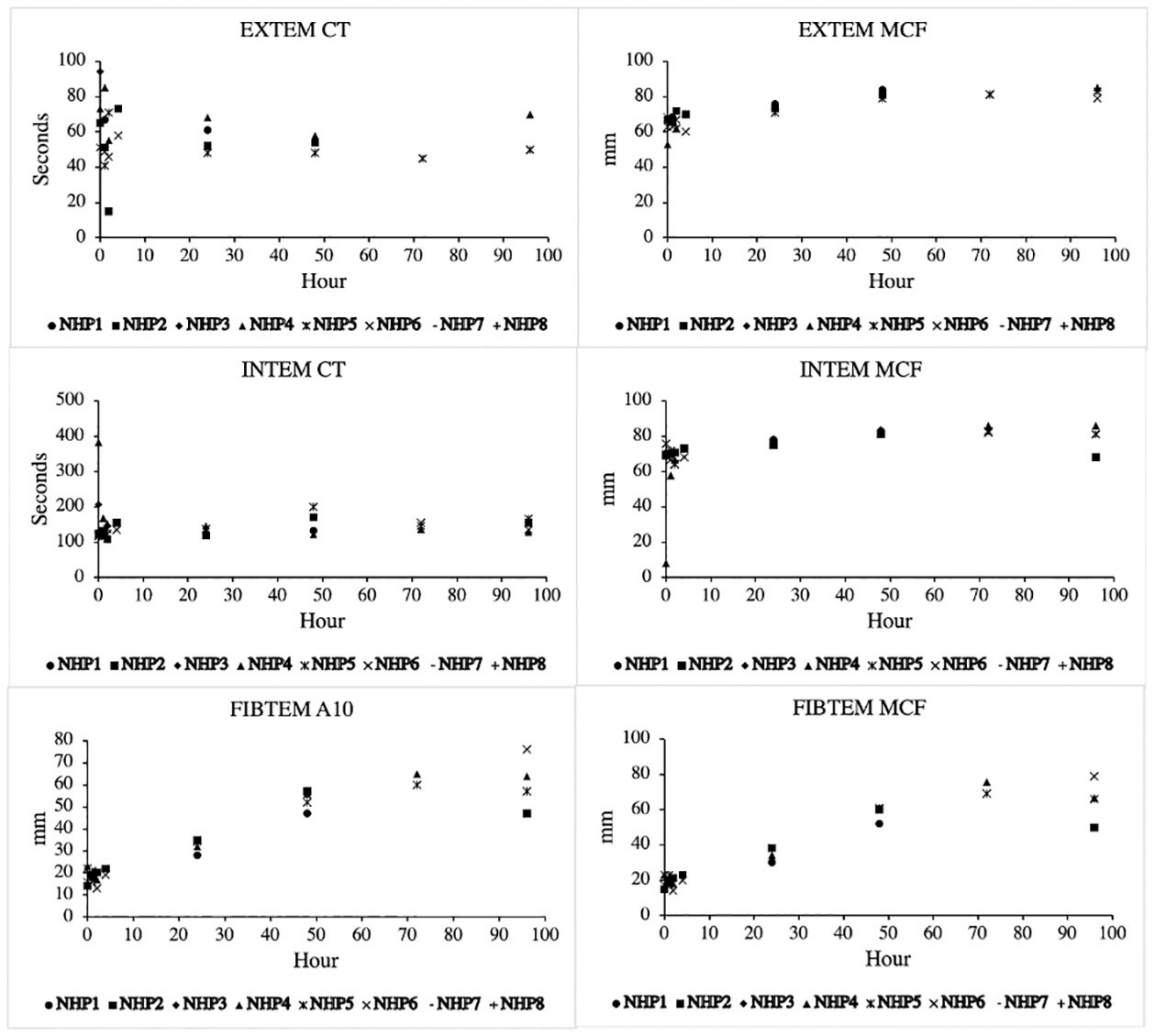
Selected ROTEM results. * denotes statistical significance from baseline values (p < 0.05).

ROTEM data is depicted in Fig 4. The intrinsic and extrinsic pathway maximum clot formation (MCF) strength and clotting time (CT) remained relatively constant throughout the experiment. Notably, the fibrin pathway amplitude (A10) and MCF increased throughout the experiment, suggesting hyperfibrinogenemia in all animals.

## Discussion

A prolonged critical care environment with ventilation and sedation is feasible but requires a significant amount of resources, time, and skill. The NHP inflammatory profile does not appear to be significantly altered by prolonged ventilation and sedation. However, NHPs may develop hyperfibrinogenemia, and pulmonary complications remain a challenging aspect of care.

Nonhuman primates imitate the human inflammatory response to sepsis or trauma, and share similar anatomy, making them ideal for animal modeling [4, 10, 13, 14, 20, 24]. Our group has previously reported multiple animal studies and characterized a Rhesus macaque inflammatory group profile in response to trauma and septic stimuli [3–5, 22]. Other studies have also characterized NHP response to insults such as resuscitative balloon occlusion of the aorta (REBOA), hemorrhage, sepsis, and spinal cord injury, but no reported trauma study has included an ICU phase with ventilatory support [13–17, 19, 25–27]. Poliquin and colleagues have recently described an Ebola virus disease model that included an ICU phase with ventilator support for Rhesus monkeys [20, 21]. NHPs serve as a useful biological surrogate for human disease processes, but meticulous and reproducible utilization of this scarce resource is crucial to the scientific community [28].

Mitigating the immunologic response to traumatic injury has led to interest in studying the prognostic value of various biomarkers, as well as targeted therapeutics to mitigate the effects of well-described relevant biomarkers [10, 11]. As traumatic models continue to evolve and require the use of a clinically relevant prolonged ICU phase, it is important to elicit the effect of non-traumatic interventions (i.e. ventilation, arteriovenous access, sedation). We found that in general, white blood cell count tended to peak early and return to baseline at about 24 hours. Transaminitis uptrended throughout the study phase, which likely indicated a hypermetabolic state, fasting, or drug (e.g. propofol) effect. We did observe that the FIBTEM A10 and MCF values increased throughout the experiment, which suggests hyperfibrinogenemia and susceptibility to venous thromboembolism which is inherent to prolonged immobilization or inflammatory state. Biomarkers did not demonstrate the dramatic fold-change that we have observed in our traumatic models. For example, the average maximum fold change for IL-1ra and IL-6 for any NHP was 224 and 15, respectively. Both fold changes were strongly attributable to the two NHPs that developed pulmonary compromise and underwent unplanned euthanasia. This in stark contrast to the average maximum fold change for IL-1ra and IL-6 in our previously reported polytrauma and hemorrhage model, which was 2286 and 785, respectively [5]. Both IL-1ra and IL-6 are potent acute phase reactants and are known to be elevated in the presence of acute lung pathologies, which was demonstrated in our NHPs that developed pulmonary complications [4, 5, 27]. The basic inflammatory bioprofile does not appear to be dramatically altered by ventilation and sedation alone, especially not near to the same order as previously described trauma models.

Our nonhuman primate intensive care unit was designed to imitate a modern trauma intensive care environment. Constant bedside presence of a certified animal care handler with physician oversight enabled us to adapt the sedation regimens, perform care tasks, and monitor the animal. The environment was carefully temperature controlled, the NHP was kept adequately padded, turned periodically to prevent ulceration, and oral care and hygiene (e.g. suctioning) was performed frequently.

We found that multiple intravenous regimens provided adequate sedation with analgesic properties necessary for an injury model, without the use of any inhalational anesthetic. These regimens included fentanyl and propofol, ketamine and xylazine, and alfaxalone and fentanyl. We did observe ketamine-induced pulmonary secretions as well as a saturable response to propofol which required the titration of a fentanyl adjunct. Several propofol-sedated animals manifested elevated blood cholesterol and triglycerides, which improved with down titration of propofol. We ultimately found that an alfaxalone and fentanyl regimen provided excellent sedation and analgesia, with more “physiologic vitals,” and without the same level of transaminitis and relative bradycardia observed with propofol and fentanyl. We found that even moderate sedation with propofol and fentanyl resulted in relative bradycardia (< 100 beats per minute) and bradypnea (< 20 breaths per minute). Notably, relative bradycardia results in decreased preload and cardiac output in NHPs [29]. Our group switched to this combination for the final two NHPs. Short-term NHP studies have shown that at low to moderate doses, alfaxalone preserves heart rate and mean arterial pressure, while producing effective sedation and analgesia [30–32]. Alfaxalone does appear to reduce cerebral blood flow and contribute to hypothermia, with higher doses also exerting suppressive cardiovascular effects [30–32].

We discovered that individual NHPs were highly sensitive to ventilation mechanics. Ultimately, our standard care “bundle” included elevating the head of the bed, aggressive inline and subglottic suctioning, and oral care with chlorhexidine. Optimal ventilator settings included PEEP 5–8 cmH2O, tidal volumes of 8 ml/kg, and a respiratory rate of 15–25. We noted that NHPs demonstrated higher pulmonary pressures (up to 25 cmH2O) when they were allowed to overbreathe the ventilator with more physiologic respiratory rates (25–30 breaths/min). Our group used the anesthesia machine ventilator for the majority of the development phase but transitioned the final two NHPs to the Drager ventilator. This enabled the use of more accurate pressure and volume curves, the ability to record the entire respiratory profile from the experiment, as well as the flexibility to add different ventilator modes not included on standard anesthesia machines.

We performed groin cutdowns for our central and arterial line insertions. The arteries were extremely spastic, and vasoconstriction around the neonatal-sized lines occasionally led to loss of distal pulse and catheter thrombosis. To mitigate this effect, we would monitor the arterial line extremity with a toe pulse oximeter, warm the extremity with a forced-air system, and frequently flush the catheter with heparinized saline. We preferred to use 22-gauge catheters. Reducing catheter size and indwelling duration are modifiable factors to reduce the risk of arterial line complications [33, 34].

The evolution of this model led to experimental refinements, adjustment of ventilator strategy, and sedation regimens. After these initial eight NHP subjects, we have continued to use this protocol and recently been able to provide consistent critical care of ventilated NHPs for 5 days even after incorporating true traumatic injury. The development of this model has allowed for the pursuance of additional work examining the prolonged (>72hrs) effects of trauma and injury on the inflammatory response.

Limitations of this study include sample rationing and nonuniformity of blood samples which made it impossible to stratify this study population into two groups. Small study population, resource rationing, and NHP and experimental heterogeneity impose inherent limitations and applicability of our findings. While experimental heterogeneity was present as we developed and refined our protocol, all eight NHPs were intubated and sedated for at least 48 hours, and no NHP underwent a traumatic injury. Thus, we believe our findings prove useful to the realm of NHP research.

## Conclusions

A NHP critical care environment with ventilation support is feasible but requires robust resources. The NHP inflammatory profile does not appear to be significantly altered during the early periods of a critical care phase with mechanical ventilation or critical care procedures. Pulmonary complications are a challenging aspect of care.

## Supporting information

S1 Checklist The ARRIVE guidelines 2.0: Author checklist. (PDF)
